# Molecular Characterization of 18S rDNA, ITS-1, ITS-2, and COI from *Eimeria christenseni* and *E. arloingi* in Goats from Shaanxi Province, Northwestern China

**DOI:** 10.3390/ani12111340

**Published:** 2022-05-24

**Authors:** Gaoxing Liang, Xin Yang, Ding Liu, Yuan Li, Junwei Wang, Xi Chen, Guanghui Zhao, Junke Song

**Affiliations:** College of Veterinary Medicine, Northwest A&F University, Xianyang 712100, China; lgx20220425@163.com (G.L.); xinyang@nwafu.edu.cn (X.Y.); ld1193@nwafu.edu.cn (D.L.); liyy0915@nwafu.edu.cn (Y.L.); wjunwei@nwafu.edu.cn (J.W.); chenxi3517@nwafu.edu.cn (X.C.)

**Keywords:** goats, *Eimeria arloingi*, *Eimeria christenseni*, 18S rDNA, ITS, COI

## Abstract

**Simple Summary:**

This study first determined the 18S rDNA, ITS-1, ITS-2, and COI sequences of *E. christenseni* and *E. arloingi*. The lengths of the 18S rDNA, ITS-1, ITS-2, and COI were 1790 bp, 403 bp, 584 bp, and 1268 bp for *E. arloingi* and 1796 bp, 386 bp, 565 bp, and 1268 bp for *E. christenseni*, respectively. Furthermore, phylogenetic analyses based on the 18S rDNA, ITS-1, ITS-2, and COI loci were conducted to assess the relationship of *E. christenseni*, *E. arloingi*, and other *Eimeria* species in ruminants.

**Abstract:**

Coccidiosis caused by *Eimeria* is one of the most common and significant diseases in goats, leading to serious economic losses in the development of the goat industry. Although several genetic loci, such as 18S rDNA, ITS-1, ITS-2, and COI, have been applied in the molecular characterization of *Eimeria* in chicken, rabbits, turkey, and wildlife, little is known about these molecular markers of *Eimeria* in goats. In the present study, we isolated purified oocysts of highly pathogenic *Eimeria* *arloingi* and *Eimeria christenseni* from fecal samples of goats in Shaanxi province, China, and then subjected these purified oocysts to genomic DNA isolation, PCR amplification, and sequencing of 18S rDNA, ITS-1, ITS-2, and COI loci of *Eimeria arloingi* and *Eimeria christenseni*. Finally, the obtained sequences were used for phylogenetic analysis of *Eimeria* species in goats and other livestock. The lengths of the 18S rDNA, ITS-1, ITS-2, and COI were 1790 bp, 403 bp, 584 bp, and 1268 bp for *E. arloingi* and 1796 bp, 386 bp, 565 bp, and 1268 bp for *E. christenseni*, respectively. The phylogenetical analysis based on 18S rDNA indicated that *E. christenseni* and *E. arloingi* were the most closely related to ovine *Eimeria*, followed by *E. bovis*, *E. ellipsoidalis*, and *E. zuernii* from cattle. The phylogenetical analysis based on ITS-1 and ITS-2 could not effectively distinguish ovine *Eimeria* from caprine *Eimeria*. The phylogenetical analysis based on the COI locus could effectively distinguish between *Eimeria* species from goats and cattle, but it was ineffective in distinguishing between *Eimeria* species from sheep and goats. To the best of our knowledge, this is the first characterization of 18S rDNA, ITS-1, ITS-2, and COI in *E. arloingi* and *E. christenseni*; it can provide useful genetic markers for molecular epidemiological and population genetic studies on *E. arloingi* and *E. christenseni* in goats and contribute to the prevention and control of goat coccidiosis.

## 1. Introduction

Coccidiosis is an important protozoosis caused by *Eimeria* spp., which mainly parasitizes the intestinal tract of various animals, including mammals, birds, reptiles, fish, and amphibians [1,2,3,4]. Recently, China became the country with the largest number of goats [5]. Coccidiosis, a common and significant disease in goats, has been widely reported in China [6,7,8]. A recent meta-analysis and systematic review indicated a total prevalence of 78.7% for *Eimeria* spp. in goats in China. A total of 12 species were identified in goats in China: *E. arloingi* (49.7%, 95% CI: 34.83–64.49), *E. alijevi* (43.7%, 95% CI: 29.53–58.45), *E. christenseni* (41.2%, 95% CI: 27.07–56.16), *E. hirci* (37.8%, 95% CI: 22.95–53.88), *E. caprina* (36.6%, 95% CI: 21.18–53.44), *E. ninakohlyakimovae* (35.9%, 95% CI: 21.02–52.31), *E. jolchijevi* (16.3%, 95% CI: 8.09–26.49), *E. pallida* (13.6%, 95% CI: 0.00–57.71), *E. caprovina* (12.1%, 95% CI: 4.92–21.6), *E. apsheronica* (9.71%, 95% CI: 5.21–15.35), *E. kocharli* 6.9% (3.01–12.16), and *E. punctata* (3.0%, 95% CI: 0.40–7.34) [8]. A previous paper reported that *Eimeria* oocysts were found in 97.3% of 584 fecal samples of goats in an epidemiological study in Shaanxi province, with six species recognized, namely *E. jolchijevi*, *E. arloingi*, *E. alijevi*, *E. caprina*, *E. hirci*, and *E. christenseni*. Among these six species, *E. arloingi* was the dominant species, with prevalences of 83.3% and 84.4% in Saanen and Guanzhong dairy goats. The prevalences were 81.6%, 68.4%, 51.7%, 29.5%, and 26.9% for *E. alijevi*, *E. jolchijevi*, *E. caprina*, *E. hirci*, and *E. christenseni* in Saanen dairy goats and 65.9%, 57.5%, 45.2%, 28.4%, and 20.7% for *E. alijevi*, *E. jolchijevi*, *E. caprina*, *E. christenseni*, and *E. hirci* in Guanzhong dairy goats, respectively [7]. The clinical symptoms of goats infected with coccidia include diarrhea, poor weight gain, weakness, and even death (particularly in kids), which significantly restrict the development of the goat industry [9,10].

Usually, host adaptations exist for the distribution of *Eimeria* species among animals. *Eimeria arloingi* and *E.* *christenseni* are currently regarded as the most highly pathogenic species among therecognized *Eimeria* species in goats [11,12,13]. Because of the differences in pathogenicity and drug resistance, rapid and accurate identification of *Eimeria* species is necessary for the effective prevention and treatment of coccidiosis in goats [9]. Traditionally, morphological observation of oocysts has been frequently used to identify species within caprine *Eimeria*; however, this approach is time-consuming, lacks specificity, and requires well-trained researchers [14]. To overcome the limitations of traditional morphological approaches, the molecular characterization of *Eimeria* species has been reported in Australia, India, Iran, Myanmar, and Oman and has proven to be useful to identify and classify these parasites, contributing to the understanding of the phylogenetic relationship among *Eimeria* species [15,16,17,18,19].

Recently, several genetic markers have been applied in the molecular characterization of *Eimeria*. One of the most common loci is the 18S rDNA, which has been used to define the phylogenetic relationship, inter- and intra-species variation among some *Eimeria* isolates [20]. Furthermore, the internal transcribed spacer 1 (ITS-1) region has also been used to establish molecular identification methods for *Eimeria* species from chicken and rabbits and has proved to be effective in clinical applications [21,22]. The mitochondrial cytochrome oxidase (COI) gene locus, which is present in many apicomplexan parasites, has also been used extensively for the identification and genotyping of species, including *Eimeria* species from turkey, ferret-badger and skink [18,23,24,25]. However, little is known about the genetic characteristics of these loci in *Eimeria* species from small ruminants, which limits the application of molecular biology techniques in the accurate classification of *Eimeria* species.

In this study, genomic DNA was isolated from purified *E. christenseni* oocysts and *E. arloingi* oocysts in goats and was then subjected to PCR amplification and sequencing of 18S rDNA, ITS-1, ITS-2, and COI loci. The obtained sequences of the four loci were subsequently used in phylogenetic analyses of *Eimeria* species. The results of the present study could contribute to the species identification, molecular epidemiology, and phylogenetic analyses of *Eimeria* species.

## 2. Materials and Methods

### 2.1. Sample Collection

From September 2020 to February 2022, a total of 245 fecal samples were collected directly from the rectums of Saanen dairy goats aged <8 months on Farm SZ in Shaanxi province, northwestern China (Table 1). Farm SZ (105°29′ E, 31°42′ N) was a captive breeding farm with over 400 Saanen dairy goats, and no drugs were applied for deworming. The fecal samples were placed into separate bags marked with basic information, transported to the lab under cool conditions, and stored at 4 °C until use.

### 2.2. Conventional Fecal Examination of Eimeria Species

The coccidial infection of each fecal sample was examined by the flotation technique using saturated sodium chloride, with coccidian oocysts per gram (OPG) quantified using a modified McMaster technique. All samples of each age group were mixed with equal quality (2 g), homogenized in saturated sodium chloride, and centrifuged at 549× *g* for 5 min. The oocysts were isolated from the supernatant and transferred into a 2.5% (*w*/*v*) aqueous potassium dichromate (K_2_Cr_2_O_7_) solution for sporulation at 28 °C. Then, the sporified oocysts of each group were identified at the species level on the basis of the time of sporulation, oocyst size, and morphological characteristics (e.g., shape, color, form index, the presence or absence of the micropyle and its cap, and the presence or absence of residual, polar, and Stieda bodies) of the oocysts and sporocysts under 400 × magnification, as previously reported, and the proportion of each *Eimeria* species in the total examined sporified oocysts was counted as the species composition (%) [26].

### 2.3. Isolation of Oocysts for E. arloingi and E. christenseni

A total of nine species were found in sporulated oocysts in goats (Table 1). The sporulated oocysts of each group were mixed and diluted with 1× PBS (phosphate-buffered saline) and then added into the McMaster egg slide counting chamber for the isolation of purified oocysts for *E. arloingi* and *E. christenseni* using a modified disposable micro-blood sampling straw. Briefly, one end of the disposable micro-blood sampling straw (Zibo Laixu Medical Instrument Co., LTD, Zibo, China) was heated using a spirit lamp to produce a thinner tip. A single oocyst from the McMaster egg slide counting chamber was isolated under 100× magnification using the modified disposable micro-blood sampling straw. Oocysts from the same species were collected in a clean PCR tube. Finally, 50 *E. christenseni* oocysts and 50 *E. arloingi* oocysts were isolated and collected in two separate PCR tubes for DNA extraction.

### 2.4. DNA Extraction

PCR tubes containing purified oocysts from *E. christenseni* and *E. arloingi* were briefly centrifuged and subjected to repeated freezing and thawing four times to disrupt the oocyst walls. Then, genomic DNA was extracted from the oocysts using DNeasy^®^ PowerSoil^®^ Pro Kit soil Genome Extraction Kit (Cat. No. 47014, Qiagen, Dusseldorf, Germany) according to the manufacturer’s instructions, and the extracted DNA (100 μL) was stored at −20 °C for future use.

### 2.5. PCR Amplification

The 18S rDNA, ITS, and COI sequence for *E. christenseni* and *E. arloingi* were obtained by PCR using general primers as reported (Table 2). PCR (25 μL) was conducted in a reaction mixture containing 1× *Ex Taq* buffer (Mg^2+^ free), 2 mM MgCl_2_, a 0.2 mM dNTP mixture, 0.625 U TaKaRa *Ex Taq*, 0.4 μΜ concentrations of each primer, and 2 μL gDNA under the following conditions: initial denaturing at 94 °C for 5 min followed by 35 cycles of denaturing at 94 °C for 45 s; annealing at 57 °C (18S rDNA), 52 °C (ITS), or 49 °C (COI) for 30 s; extension at for 72 °C for 90 s; and a final extension at 72 °C for 10 min. All amplicons were examined by electrophoresis in 1% agarose gel with ethidium bromide, and positive amplicons were purified using the Universal DNA Purification Kit, cloned into the pMD19-T vector, and transformed into *Escherichia coli* JM109. Then, the positive transformants were confirmed by PCR under the above-mentioned conditions and sequenced by Sangon Biotech (Shanghai, China) using an ABI 730 Autosequencer.

### 2.6. Sequence Analysis

All the obtained sequences were confirmed to be the corresponding loci using BLAST alignment from NCBI (https://blast.ncbi.nlm.nih.gov/Blast.cgi, accessed date: 20 May 2021) and aligned with reference sequences downloaded from GenBank using Clustal X 1.83 (ftp://ftpigbmc.u-strasbg.fr/pub/ClustalX/, accessed date: 25 May 2021). To identify the phylogenetic relationships of *E. christenseni* and *E. arloingi* with selected *Eimeria* species, four trees based on 18S rDNA, ITS-1, ITS-2, and COI sequences were constructed using the Neighbor-Joining (N-J) method in MEGA 6.06 [28]. The Kimurás two-parameter method was used with bootstrap evaluation of 1000 replicates.

### 2.7. Nucleotide Sequence Accession Numbers

The representative nucleotide sequences of 18S rDNA, ITS-1, ITS-2, and COI of *E. christenseni* and *E. arloingi* were submitted to the GenBank database under the accession numbers ON259585–ON259586, ON261603–ON261604, ON261608–ON261609 and ON245513–ON245514.

## 3. Results

### 3.1. Conventional Fecal Examination of Eimeria Species

**Figure 1:**
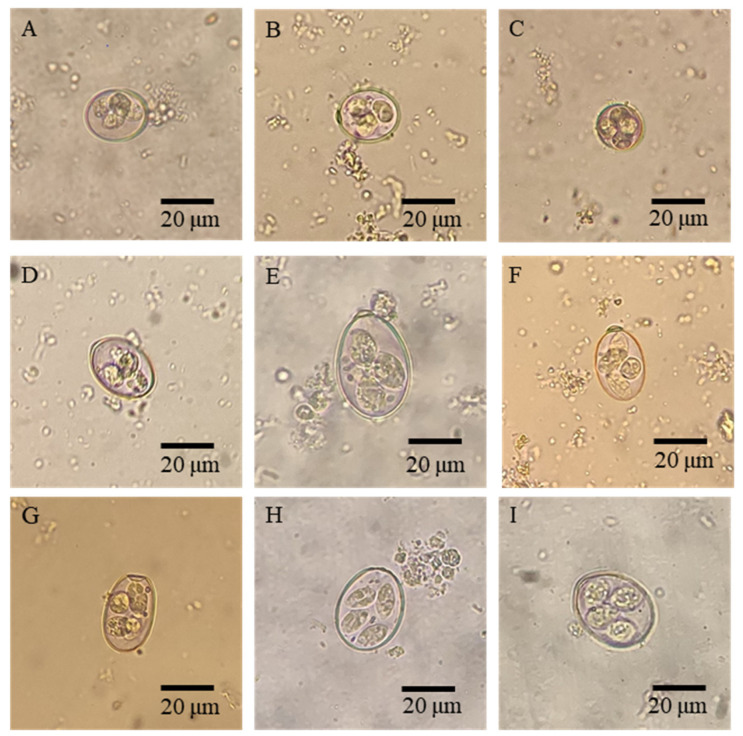
Morphological examination of sporulated oocysts of nine *Eimeria* species identified in goats in the present study. (**A**–**I**) Sporulated oocysts of *E. ninakohlyakimovae*, *E. hirci*, *E. alijevi*, *E.* *jolchijevi*, *E. christenseni*, *E. arloingi*, *E. caprina*, *E. caprovina,* and *E. apsheronica*, respectively.

The total prevalence of *Eimeria* spp. in our study was 95.92% (235/245), with the highest prevalence (100%) in goats aged 2–3 months, 4–5 months, 5–6 months, 6–7 months, and 7–8 months, while lowest prevalence (82.14%) was in goats aged 1–2 months. As for the infection intensity of *Eimeria* spp. in each age group, the highest OPG was found in goats aged 2–3 months (162,080 ± 405,852), while the lowest was found in goats aged 7–8 months (7738 ± 6229) (Table 1). Furthermore, *Eimeria* oocysts in each group were sporulated for species identification and composition analyses, and nine *Eimeria* species were identified in each age group (Table 1), namely *E. christenseni*, *E. arloingi*, *E. ninakohlyakimovae*, *E. hirci*, *E. jolchijevi*, *E. alijevi*, *E. caprina*, *E. caprovina*, and *E. apsheronica* (Figure 1), with *E. christenseni* and *E. arloingi* being the major species.

### 3.2. Characterization of 18S rDNA of E. christenseni and E. arloingi

**Figure 2:**
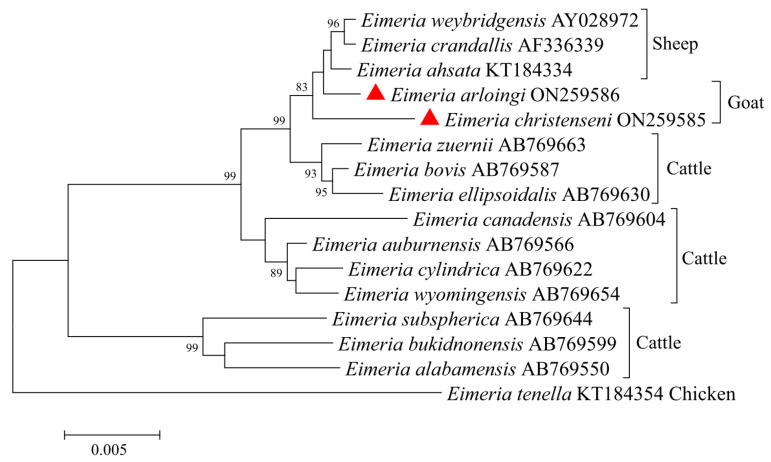
Phylogenetic tree generated by the Neighbor-Joining method using partial sequences of the 18S rDNA region of *E. christenseni* and *E. arloingi* from the present study and representative sequences of other *Eimeria* species available in GenBank. The sequences obtained in this study are marked with red triangles (bootstrap values below 75% are not indicated).

The obtained partial 18S rDNA sequences of *E. arloingi* and *E. christenseni* were 1790 bp and 1796 bp in length, respectively. Then, we used the 18S rDNA sequences of *E. arloingi*, *E. christenseni*, another 13 species from ruminant animals (*E. weybridgensis*, *E. crandallis*, *E. ahsata*, *E. zuernii*, *E. bovis*, *E. ellipsoidalis*, *E. canadensis*, *E. auburnensis*, *E. cylindrica*, *E. wyomingensis*, *E. subspherica*, *E. bukidnonensis*, and *E. alabamensis*), and *E. tenella* from chicken for a genetic relationship analysis (Figure 2). The tree consisted of two large clades with 99% support: one contained three, two, and seven species from sheep, goats, and cattle, respectively; the other contained three species from cattle. *E. arloingi* and *E. christenseni* from goats in this study were included in the same branch with species from sheep and had a relatively close relationship with *E. weybridgensis*, *E. crandallis*, and *E. ahsata* from sheep, followed by species from cattle.

### 3.3. Characterization of ITS-1 of E. christenseni and E. arloingi

**Figure 3:**
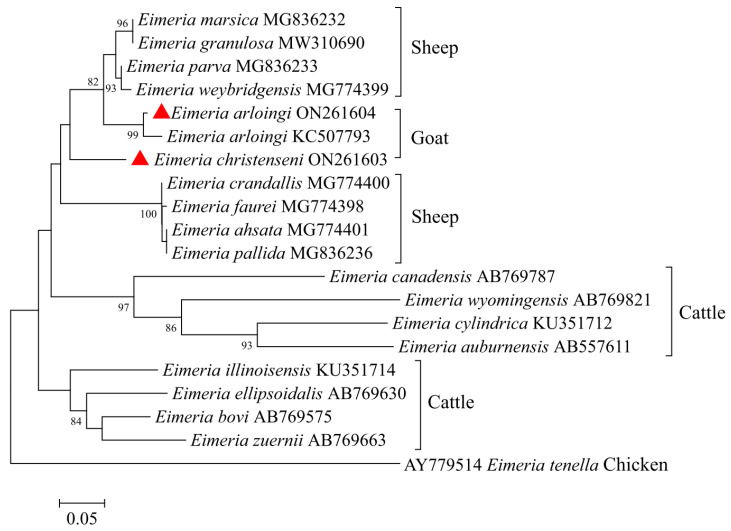
Phylogenetic tree generated by the Neighbor-Joining method using full sequences of the ITS-1 region of *E. christenseni* and *E. arloingi* from the present study and representative sequences of other *Eimeria* species available in GenBank. The sequences obtained in this study are marked with red triangles (bootstrap values below 75% are not indicated).

The full ITS-1 sequences of *E. arloingi* and *E. christenseni* were 403 bp and 386 bp in length, respectively. Subsequently, we used the ITS-1 sequences of *E. arloingi*, *E. christenseni*, another 17 selected ruminant species, and one chicken species (*E. tenella*) for a genetic relationship analysis (Figure 3). The tree consisted of three large clades: one contained eight and three species from sheep and goats, respectively, and the other two clades contained four cattle species each. *E. arloingi* and *E. christenseni* from goats in this study had a relatively close relationship with *E. arloingi* (KC507793) from goats, followed by species from sheep and cattle.

### 3.4. Characterization of ITS-2 of E. christenseni and E. arloingi

**Figure 4:**
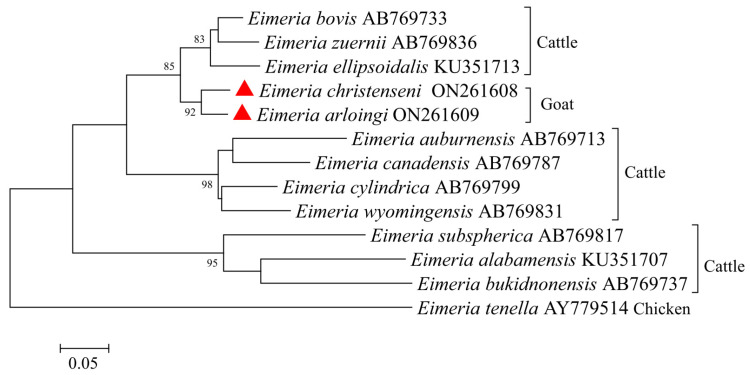
Phylogenetic tree generated by the Neighbor-Joining method using full sequences of the ITS-2 region of *E. christenseni* and *E. arloingi* from the present study and representative sequences of other *Eimeria* species available in GenBank. The sequences obtained in this study are marked with red triangles (bootstrap values below 75% are not indicated).

The full ITS-2 sequences of *E. arloingi* and *E. christenseni* were 584 bp and 565 bp in length, respectively. Then, we used the ITS-2 sequences of *E. arloingi*, *E. christenseni*, another 10 selected ruminant species, and one chicken species *(E. tenella*) for a genetic relationship analysis (Figure 4). The tree consisted of two large clades: one contained seven and two species from cattle and goats, respectively, and the other contained three species from cattle. *E. arloingi* and *E. christenseni* from goats in this study were included in one separate branch and had a relatively close relationship with *E. bovis*, *E. zuernii*, and *E. ellipsoidalis* from cattle.

### 3.5. Characterization of COI of E. christenseni and E. arloingi

**Figure 5:**
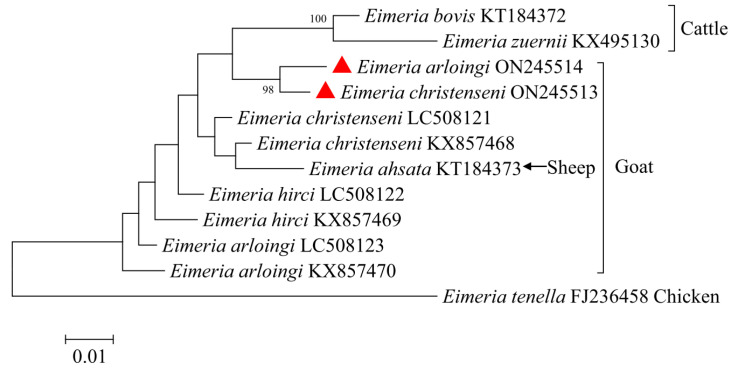
Phylogenetic tree generated by the Neighbor-Joining method using partial sequences of the COI region of *E. christenseni* and *E. arloingi* from the present study and representative sequences of other *Eimeria* species available in GenBank. The sequences obtained in this study are marked with red triangles (bootstrap values below 75% are not indicated).

The partial COI sequences of both *E. arloingi* and *E. christenseni* were 1268 bp in length. Subsequently, we used the COI sequences of *E. arloingi*, *E. christenseni*, another six selected ruminant species, and one chicken species (*E. tenella*) for a genetic relationship analysis (Figure 5). *E. arloingi* and *E. christenseni* from goats in this study were included in one separate branch and had a relatively close relationship with *E. bovis* and *E. zuernii* from cattle, followed by species from sheep and goats. The comparative analysis results of the similarities between the COI sequences showed that the percent identity among *Eimeria* species from sheep and goats was 94.4–98.9%, and the percent identity between *E. ahsata* from sheep and other *Eimeria* species from goats was 95.3–98.3% (Table 3).

## 4. Discussion

Goat farming is an important part of animal husbandry in China [5]. At present, goat coccidiosis seriously affects the milk, meat, and other production traits of goats, causing huge economic losses in the world; goat coccidiosis has become one of the problems hindering the healthy development of the goat farming industry [13]. The present study first determined the 18S rDNA, ITS-1, ITS-2, and COI sequences of *E. arloingi* and *E. christenseni* and analyzed the phylogenetic relationship of *E. arloingi*, *E. christenseni*, and other reported representative *Eimeria* species available in GenBank, which could contribute to the understanding of the biology, molecular epidemiology, and population diversity of *Eimeria* species. In addition, it will also help support the host adaptation of *Eimeria* species of different animals, including sheep, goats, and cattle.

The 18S rDNA locus has been used extensively as a molecular marker in phylogenetic analysis. In the present study, the phylogenetic tree based on the 18S rDNA sequence showed that *E. christenseni* and *E. arloingi* were the most closely related to ovine *Eimeria*, followed by *E. bovis*, *E. ellipsoidalis*, and *E. zuernii* from cattle. Unfortunately, caprine *Eimeria* and ovine *Eimeria* were not effectively distinguished. Since the 18S rDNA is a conservative gene among *Eimeria* species, it is better to combine it with other gene loci (e.g., ITS-1, ITS-2, and COI) to comprehensively understand their phylogenetic relationship [15,19,20].

The ITS-1 locus is also a common marker for phylogenetic analysis [22]. In this study, although caprine *Eimeria* (*E. arloingi* and *E. christenseni*) were placed on two separate branches in the phylogenetic tree based on the ITS-1 region, they may not be effectively distinguished from ovine *Eimeria* because of low bootstrap values. Hence, it is suggested that the ITS-1 sequence may not be a useful marker for the understanding of phylogenetic relationships among *Eimeria* species from ruminants. Similarly, the ITS-2 locus may also not be a suitable marker to reveal the relationships among *Eimeria* species, but this needs to be verified in more sequences.

Partial COI sequences could provide more sufficient variability, which is needed to distinguish species efficiently, than complete 18S rDNA sequences from the same taxa using different *Eimeria* species from chicken [20]. In this study, although the COI locus could effectively distinguish *Eimeria* species from goats and cattle, it was ineffective in distinguishing between *Eimeria* species from sheep and goats, primarily because of the low resolution achieved here using a partial sequence of ~640 bp.

Now that the 18S rDNA, ITS-1, ITS-2, and COI sequences of two highly pathogenic *Eimeria* species in goats are available, it will be interesting to conduct comprehensive studies to understand the biology, epidemiology, and population diversity of these *Eimeria* species by integrating conventional morphological examination with PCR analysis. Notably, multiple loci with high resolution are needed in the phylogenetic analyses of *Eimeria* species in ruminants instead of only one locus.

## 5. Conclusions

The total prevalence of *Eimeria* spp. was 95.92% in goats in the present study with nine species recognized, namely *E. christenseni*, *E. arloingi*, *E. ninakohlyakimovae*, *E. alijevi*, *E. jolchijevi*, *E. hirci*, *E. caprina*, *E. apsheronica*, and *E. caprovina*. This is the first report of 18S rDNA, ITS-1, ITS-2, and COI sequences of *E. christenseni* and *E. arloingi*. The obtained sequences should be useful as sources for genetic markers for molecular epidemiological and population genetic studies on *E. christenseni* and *E. arloingi* in goats.

## Figures and Tables

**Table 1:** Occurrence of *Eimeria* spp. and species composition in goats on Farm SZ.

Age	No. Tested	No. Positive (%)	OPG (Mean ± SD)	Species Composition (%)									
				ECH ^a^	EAR ^b^	ENI ^c^	EAL ^d^	EJO ^e^	EHI ^f^	ECI ^g^	EAP ^h^	ECO ^i^	Others
<1 month	13	11 (84.62)	8739 ± 18,778	6.11	40.46	28.24	5.34	0.00	16.79	1.53	1.53	0.00	0.00
1–2 months	28	23 (82.14)	7989 ± 13,430	41.98	26.72	8.40	1.53	0.76	15.27	3.05	5.34	1.53	0.76
2–3 months	39	39 (100)	162,080 ± 405,852	37.86	12.86	15.71	4.29	0.00	12.14	2.14	5.71	7.14	2.14
3–4 months	76	73 (96.05)	82,345 ± 341,874	17.07	28.46	17.89	4.88	1.63	17.89	2.44	2.44	6.50	0.81
4–5 months	33	33 (100)	86,497 ± 165,841	29.29	23.57	18.57	3.57	0.71	9.29	3.57	4.29	6.43	0.71
5–6 months	12	12 (100)	27,725 ± 26,902	36.43	10.00	12.14	2.14	0.71	5.71	2.14	5.00	5.00	2.14
6–7 months	20	20 (100)	11,290 ± 9967	24.29	23.57	11.43	4.29	0.00	3.57	1.43	2.14	4.29	0.71
7–8 months	24	24 (100)	7738 ± 6229	0.00	42.15	22.31	6.61	4.13	15.70	1.65	3.31	3.31	0.83
Total	245	235 (95.92)	67,409 ± 260,945	24.13	25.97	16.84	4.08	0.99	12.05	2.24	3.72	4.27	1.01

^a/b/c/d/e/f/g/h/i^*E. christenseni/E. arloingi/E. ninakohlyakimovae/E. alijevi/E. jolchijev/E. hirci/E. caprina/E. apsheronica/E. caprovina*.

**Table 2:** Primers used in this study.

Target	Primer ID	Sequence (5′–3′)	Amplicon Size (bp)	Reference
18S rDNA	ERIB1	ACCTGGTTGATCCTGCCAG	~1790	[27]
	ERIB10	CTTCCGCAGGTTCACCTACGG		
ITS1-ITS2	ITS-1	GGATGCAAAAGTCGTAACACGG	~1010	[27]
	ITS-2	TCCTCCGCTTAATAATATGC		
COI	COI_UNI_199F	ATGATYTTCTTTGTAGTTATGCC	~1272	[24]
	mtRNA20_UNI	GTATGGATTTCACGGTCAA		

**Table 3:** Pairwise identities (%) among the COI sequences of *E. christenseni* and *E. arloingi*, *E. ahsata*, and *E. hirci*.

Sample Code		1	2	3	4	5	6	7	8	9
**1**	*E. christenseni* ON245513	-								
**2**	*E. arloingi* ON245514	98.4	-							
**3**	*E. ahsata* KT184373	96.7	96.6	-						
**4**	*E. christenseni* KX857468	96.9	96.2	98.3	-					
**5**	*E. christenseni* LC508121	96.9	96.7	97.8	98.9	-				
**6**	*E. arloingi* LC508123	95.6	95.5	96.4	97.2	98.0	-			
**7**	*E. arloingi* KX857470	94.8	94.4	95.3	96.4	96.9	98.6	-		
**8**	*E. hirci* KX857469	95.3	94.8	95.8	97.2	97.3	98.4	97.7	-	
**9**	*E. hirci* LC508122	96.6	96.4	96.9	98.0	98.8	98.4	97.3	98.4	-

## Data Availability

Data are contained within the article.
